# UBXN3B Controls Immunopathogenesis of Arthritogenic Alphaviruses by Maintaining Hematopoietic Homeostasis

**DOI:** 10.1128/mbio.02687-22

**Published:** 2022-11-15

**Authors:** Tingting Geng, Duomeng Yang, Tao Lin, Jason G. Cahoon, Penghua Wang

**Affiliations:** a Department of Immunology, School of Medicine, the University of Connecticut Health Center, Farmington, Connecticut, USA; Virginia Tech; Virginia Tech

**Keywords:** UBXN, alphavirus, Chikungunya virus, arthritogenic, hematopoiesis

## Abstract

Ubiquitin regulatory X domain-containing proteins (UBXN) might be involved in diverse cellular processes. However, their *in vivo* physiological functions remain largely elusive. We recently showed that UBXN3B positively regulated stimulator-of-interferon-genes (STING)-mediated innate immune responses to DNA viruses. Herein, we reported the essential role of UBXN3B in the control of infection and immunopathogenesis of two arthritogenic RNA viruses, Chikungunya (CHIKV) and O’nyong’nyong (ONNV) viruses. *Ubxn3b* deficient (*Ubxn3b*^−/−^) mice presented higher viral loads, more severe foot swelling and immune infiltrates, and slower clearance of viruses and resolution of inflammation than the *Ubxn3b^+/+^* littermates. While the serum cytokine levels were intact, the virus-specific immunoglobulin G and neutralizing antibody levels were lower in the *Ubxn3b*^−/−^ mice. The *Ubxn3b*^−/−^ mice had more neutrophils and macrophages, but much fewer B cells in the ipsilateral feet. Of note, this immune dysregulation was also observed in the spleens and blood of uninfected *Ubxn3b*^−/−^ mice. UBXN3B restricted CHIKV replication in a cell-intrinsic manner but independent of type I IFN signaling. These results demonstrated a dual role of UBXN3B in the maintenance of immune homeostasis and control of RNA virus replication.

## INTRODUCTION

*Alphavirus* is a genus of the *Togaviridae* family of positive sense, single-stranded RNA viruses. These viruses are primarily mosquito-borne and pose a public health threat worldwide, particularly in tropical/subtropical regions. Several of them, including Chikungunya (CHIKV), O’nyong’nyong (ONNV), and Ross River viruses (RRV), are known to elicit arthritis in humans. Of note, CHIKV causes acute and chronic crippling arthralgia and long-term neurological disorders. CHIKV was first discovered in 1952, in Tanzania and was relatively silent for several decades. However, since 2005 CHIKV has re-emerged and caused several large outbreaks in Africa, Asia, and South America (1). CHIKV appeared on the Caribbean Islands in 2013 and has spread rapidly throughout Central and South America since then, resulting in ~3 million human infections and ~500 deaths (data were from the Pan America Health Organization). CHIKV infection in humans can be divided into two phases, with an acute viremic stage for approximately 1 week and a chronic phase without active viral replication but sustained inflammation for several months to years. Both phases are typical of elevated levels of interferons (IFN) and inflammatory mediators, massive immune infiltrates primarily including macrophages and monocytes but also neutrophils (acute phase only), dendritic cells, NK cells, and lymphocytes (2–7). Approximately 50% of CHIKV-infected patients suffer from rheumatic manifestations, with ~5% of the victims suffering from chronic inflammatory rheumatism (1, 8). However, there are no approved vaccines or specific antiviral drugs. This is partly due to a poor understanding of the protective and detrimental immune responses elicited by CHIKV. In mouse models, CHIKV infection results in brief viremia for usually 5 to 7 days, which is controlled primarily by the type I interferon (IFN) response (6, 9–11). However, viral clearance depends on virus-specific antibody responses (12–19). When inoculated directly into a mouse foot, CHIKV induces the first peak of foot swelling characteristic of edema at 2 to 3 days postinfection followed by a second peak with massive immune infiltrates at 6 to 8 days postinfection (10, 20–26).

Ubiquitin regulatory X (UBX) domain-containing proteins (UBXN) are putative cofactors of a highly conserved AAA ATPase, p97/VCP, which participates in diverse cellular processes such as homotypic membrane fusion, endoplasmic reticulum (ER) associated degradation (ERAD) of misfolded proteins, cell cycle, etc. (27). UBXNs could also be adaptors interfacing E3 ubiquitin ligases and their substrates (28, 29). We and other research groups recently showed that several UBXNs regulate the immune system including viral RNA-sensing RIG-I (retinoic acid-inducible gene 1) like receptor-mitochondrial antiviral viral signaling (RLR-MAVS) (30–32), Nuclear factor-κB (NF-κB) signaling (33, 34), Janus kinase (JAK)-signal transducer and activator of transcription proteins (STAT) signaling (35), and mitophagy (36), etc. However, the *in vivo* physiological functions of UBXNs remain poorly understood. To this end, with a mouse model we recently found that UBXN3B controlled DNA virus infection by positively regulating the viral DNA-sensing, cGAS (cyclic di-GMP-AMP synthase)-STING (stimulator-of-interferon-genes) signaling and innate immune responses (37). Of note, STING is critical to the control of CHIKV infection and arthritis pathogenesis likely independently of the type I IFN response (38). Therefore, we asked if UBXN3B also played an important role in the control of CHIKV pathogenesis in mice. Our results showed that *Ubxn3b* deficient (*Ubxn3b*^−/−^) mice were more prone to CHIKV/ONNV infection than *Ubxn3b^+/+^* littermates, which was characterized by higher viral loads, more severe foot swelling and immune infiltrates, slower clearance of viruses, and resolution of inflammation. The levels of serum type I interferons (IFNs) and inflammatory mediators were similar or higher, while the serum virus-specific immunoglobulin G and neutralizing antibody levels were lower in *Ubxn3b*^−/−^. Mechanistically, UBXN3B was essential for the maintenance of immune cell homeostasis during viral infection and in steady-state. UBXN3B also controlled CHIKV replication in a cell-intrinsic but in a type I IFN-independent manner.

## RESULTS

### An essential role of UBXN3B in the control of CHIKV/ONNV pathogenesis.

**FIG 1:**
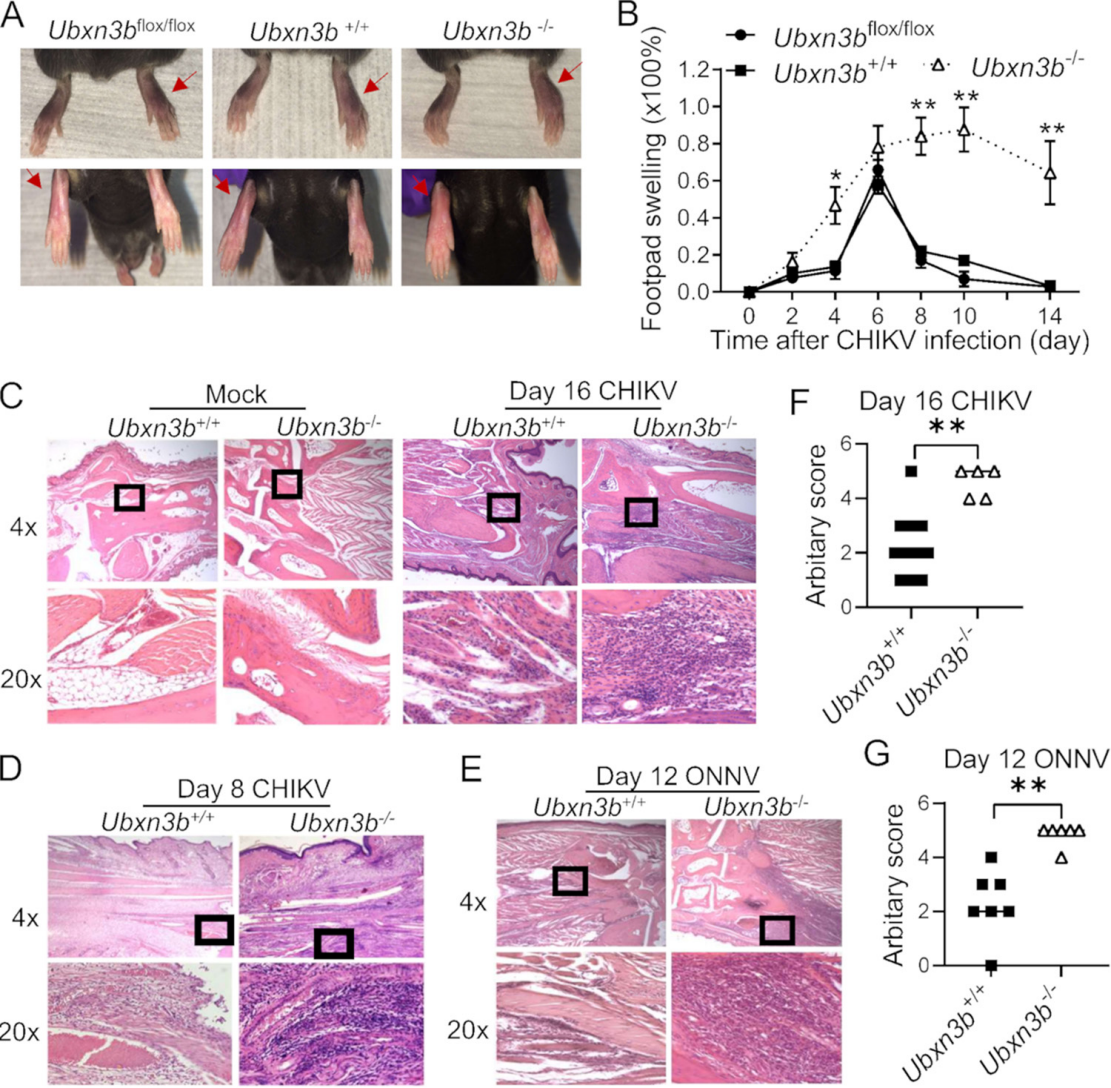
UBXN3B was critical for the control of CHIKV/ONNV arthritis. Sex-and-age matched littermates were administered 1 × 10^5^ PFU of CHIKV or 5 × 10^5^ of ONNV subcutaneously in one footpad of *Ubxn3b*^+/+^ (corn-oil treated Cre^+^ *Ubxn3b*^flox/flox^), *Ubxn3b*^−/−^ (tamoxifen-treated Cre^+^ *Ubxn3b*^flox/flox^), and *Ubxn3b*^flox/flox^ (tamoxifen-treated Cre negative *Ubxn3b*^flox/flox^, a control to exclude any effect of tamoxifen on foot swelling/viral replication) littermates. (A) Representative images of foot swelling on day 8 postinfection (pi) (*n* = 3 animals per group). The red arrows indicate the foot inoculated with CHIKV. (B) Percent increase in the footpad dimensions of infected mice over before infection (baseline). Data point, mean ± SEM (*n* = 3 mice per genotype). (C to E) Representative micrographs of hematoxylin and eosin staining of ankle joints (C) on day 16 (N = 10 *Ubxn3b*^+/+^, 5 *Ubxn3b*^−/−^animals), (D) on day 8 (N = 3/group) post-CHIKV pi, and (E) on day 12 after ONNV infection (N = 7 *Ubxn3b*^+/+^, N = 6 *Ubxn3b*^−/−^animals). Objective lens, 4× and 20×. The lower plots (C to E) are the magnified (20×) portions of the squares in the upper plots. (F and G) The arbitrary scores of foot inflammation and damage using scales of 1 to 5, with 5 representing the worst disease presentation. Each symbol is one mouse. Error bar, mean ± SEM; *, *P* < 0.05; **, *P* < 0.01 (two-tailed Student's *t* test). The results are representative of 2 independent experiments.

**FIG 2:**
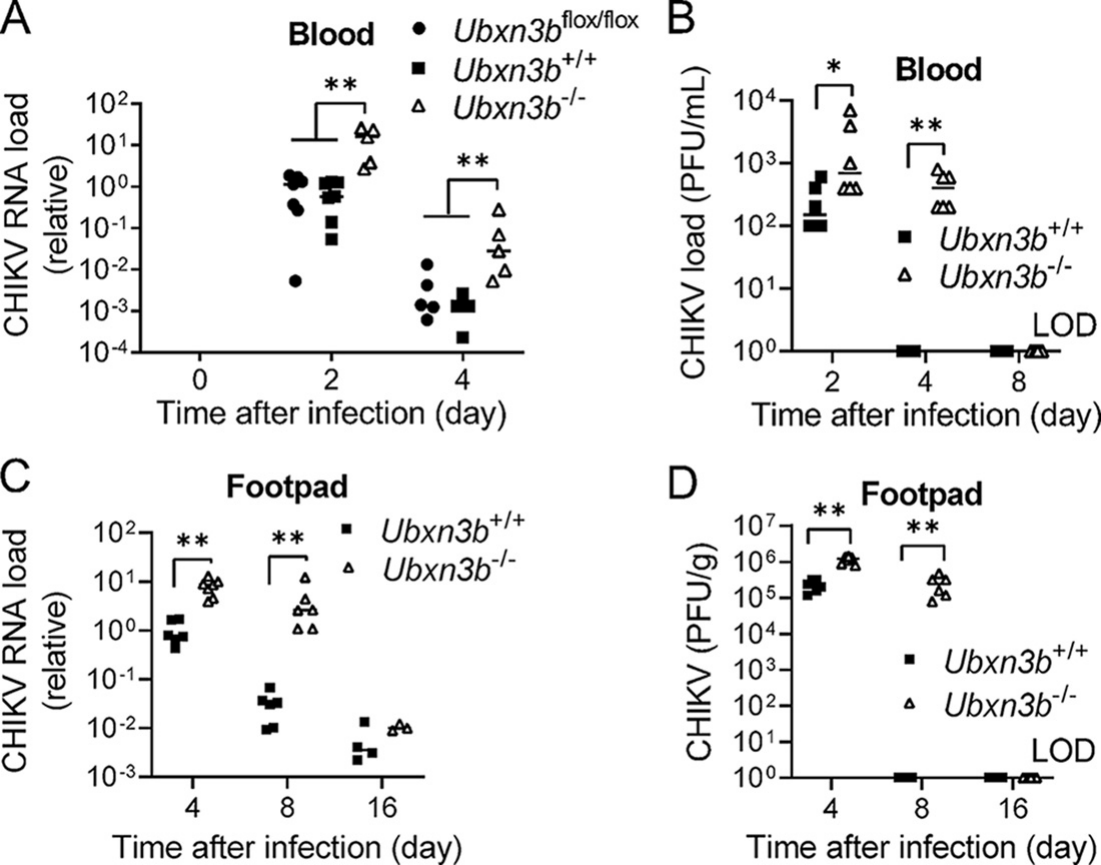
UBXN3B was essential for the control of CHIKV infection in mice. Sex-and-age matched littermates were administered 1 × 10^5^ PFU of CHIKV subcutaneously in one footpad. (A and C) Quantitative RT-PCR (qPCR) quantification of CHIKV loads in (A) whole blood and (C) feet. (B and D) Viral titers in the (B) sera (plaque forming units [PFU/mL]) and (D) feet (PFU/gram tissue). Each symbol is one mouse, N = 6/group in (B and D). The short horizontal line is the median of the result. *, *P* < 0.05; **, *P* < 0.01 (two-tailed Student's *t* test). LOD, limit of detection. The results are representative of 2 independent experiments.

We recently showed that UBXN3B controls DNA virus infection via STING (37), and STING is critical to the control of CHIKV infection and arthritis pathogenesis in mice (38). However, the UBXN3B function in RNA virus infection has not been studied in depth. To this end, we employed a tamoxifen-inducible Cre/LoxP model because UBXN3B was essential for embryonic development. We directly injected CHIKV into one footpad to induce swelling and acute arthritis in *Ubxn3b*^+/+^ (corn-oil treated Cre^+^ *Ubxn3b*^flox/flox^), *Ubxn3b*^−/−^ (tamoxifen-treated Cre^+^ *Ubxn3b*^flox/flox^), and *Ubxn3b*^flox/flox^ (tamoxifen-treated Cre negative *Ubxn3b*^flox/flox^, a control to exclude any effect of tamoxifen on foot swelling/viral replication) littermates. Both the *Ubxn3b*^+/+^ and *Ubxn3b*^flox/flox^ mice showed progressive footpad swelling, which peaked at 6 days postinfection (pi) and resolved rapidly. In contrast, the *Ubxn3b*^−/−^ mice presented more severe and persistent foot swelling at 4 days pi through 14 days pi (Fig. 1A and B). Histopathological analyses by hematoxylin and eosin (H&E) staining confirmed more immune infiltrates in the muscle and synovial cavity of *Ubxn3b*^−/−^ than those of *Ubxn3b*^+/+^ littermates at 8 and 16 days pi (Fig. 1C to E). Similar results were observed with infection of another arthritogenic alphavirus, O’nyong’nyong (ONNV) at 12 days pi (Fig. 1E and G). Moreover, the CHIKV viremia at 2 and 4 days pi, and viral loads in the ipsilateral feet of *Ubxn3b*^−/−^ mice at 4 and 8 days pi were significantly higher than those in *Ubxn3b*^+/+^ and *Ubxn3b*^flox/flox^ littermates (Fig. 2). Of note, the infectious virions were cleared from the blood by 4 days and the feet by 8 days pi in the *Ubxn3b*^+/+^ while it remained high in the *Ubxn3b*^−/−^ mice (Fig. 2B and D), suggesting a critical role of UBXN3B in the resolution of viral infection.

**FIG 3:**
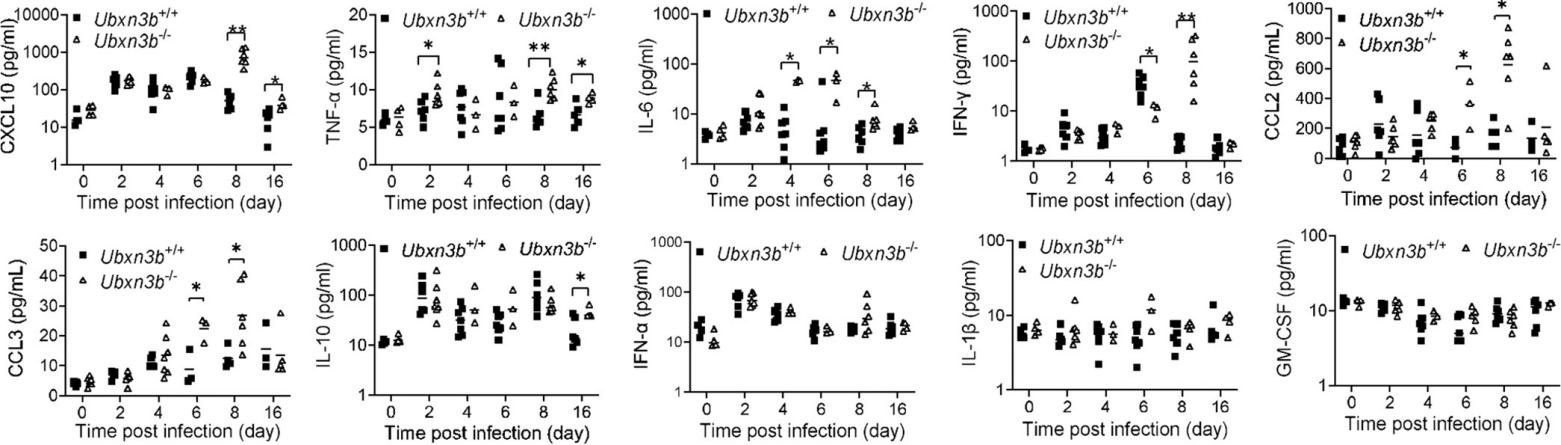
Cytokine responses were intact during CHIKV infection. Sex-and-age matched littermates were administered 1 × 10^5^ PFU of CHIKV subcutaneously in one footpad. The serum cytokines, chemokines, and growth factors were quantitated by a bead-based multiplex ELISA, except for CCL2 and CCL3, which were quantified by individual ELISA. Each symbol is one mouse. The short horizontal line is the median of the result. *, *P* < 0.05; **, *P* < 0.01 (two-tailed Student's *t* test). The results are representative of 2 independent experiments.

**FIG 4:**
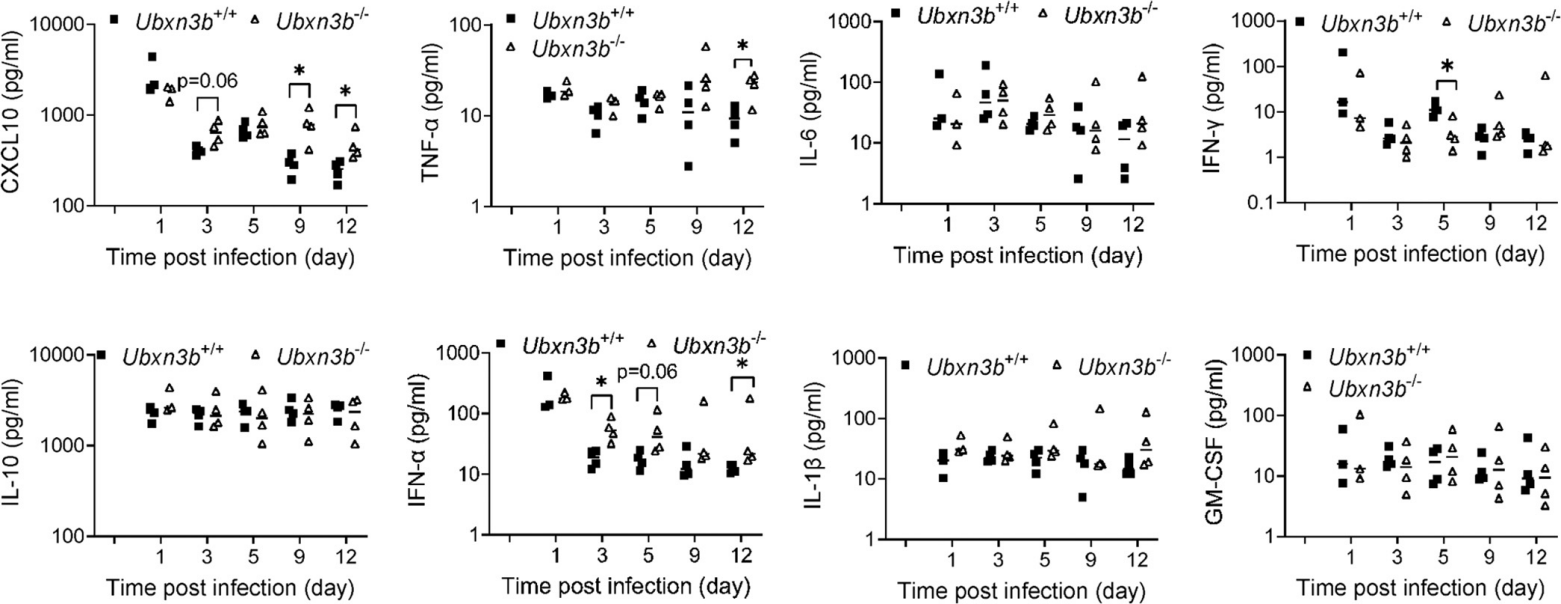
Cytokine responses were intact during ONNV infection. Sex-and-age matched littermates were administered 5 × 10^5^ PFU of ONNV subcutaneously in one footpad. Serum cytokines, chemokines, and growth factors were quantitated by a bead-based multiplex ELISA. Each symbol is one mouse. The short horizontal line is the median of the result. *, *P* < 0.05; **, *P* < 0.01 (two-tailed Student's *t* test). The results are representative of 2 independent experiments.

Acute CHIKV infection was associated with a systemic IFN and inflammatory response. Next, we measured the concentrations of serum innate immune molecules, including early antiviral type I IFNs (IFN-α) and type II IFN (IFN-γ), and those inflammatory mediators associated with severe arthritis in humans such as tumor necrosis factor (TNF-α), interleukin 6 (IL-6), IL-10, C-X-C motif chemokine ligand 10 (CXCL10), IL-1β, and granulocyte-macrophage colony-stimulating factor (GM-CSF). We noted that most cytokine levels were upregulated following CHIKV infection, though with different kinetics, except for IL-1β and GM-CSF (Fig. 3). The levels of CXCL10, TNF-α, IL-6, IL-10, CCL2, and CCL3 were higher in the *Ubxn3b*^−/−^ than those in *Ubxn3b*^+/+^ mice at one or several time points even after viremia had been cleared at 8 days pi through 16 days pi (Fig. 3). The IFN-γ level peaked on day 6 in the *Ubxn3b*^+/+^ mice, and this peak shifted to day 8 in the *Ubxn3b*^−/−^ animals, while the IFN-α level was similar between the *Ubxn3b*^−/−^ and *Ubxn3b*^+/+^ mice (Fig. 3). During ONNV infection, we observed that the CXCL10 and IFN-α levels were higher in the *Ubxn3b*^−/−^ than those in *Ubxn3b*^+/+^ littermates at several time points, and TNF-α at 12 days pi (Fig. 4). The differences between CHIKV and ONNV infection were likely due to different infection kinetics. We noted that ONNV infection caused more moderate foot swelling than CHIKV infection. Nonetheless, both the CHIKV and ONNV data demonstrated that cytokine responses were still intact or even higher in the *Ubxn3b*^−/−^ mice.

### An essential role of UBXN3B in the maintenance of immune cell homeostasis.

**FIG 5:**
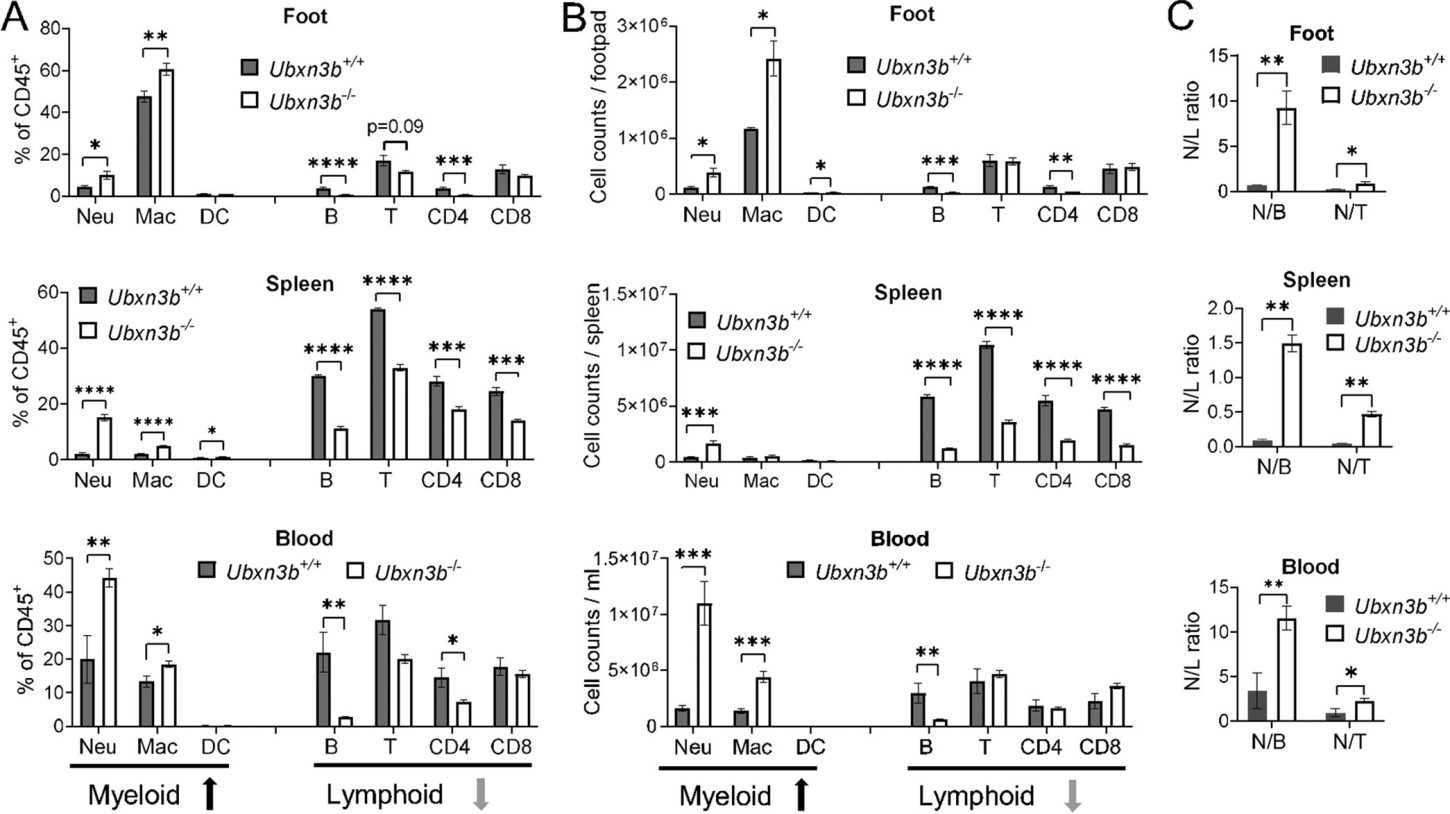
UBXN3B was essential for immune cell hemostasis during CHIKV infection. Sex-and-age matched littermates were administered 1 × 10^5^ PFU of CHIKV subcutaneously in one footpad. On day 7 after infection, various immune cell compartments in the foot, spleen, and blood were analyzed by flow cytometry. (A) The percentage relative to CD45^+^ cells, (B) the counts of each immune cell type, and (C) the neutrophil-to-lymphocyte ratios (N/L). Neu, neutrophil; Mac, macrophage; DC, dendritic cell. The red/green arrows at the bottom indicate an increase/decrease in the *Ubxn3b*^−/−^ compared to *Ubxn3b*^+/+^ mice. Bar, mean ± SEM; N = 6. *, *P* < 0.05; **, *P* < 0.01; ***, *P* < 0.001; ****, *P* < 0.0001 (two-tailed Student's *t* test). The results are representative of 2 independent experiments.

**FIG 6:**
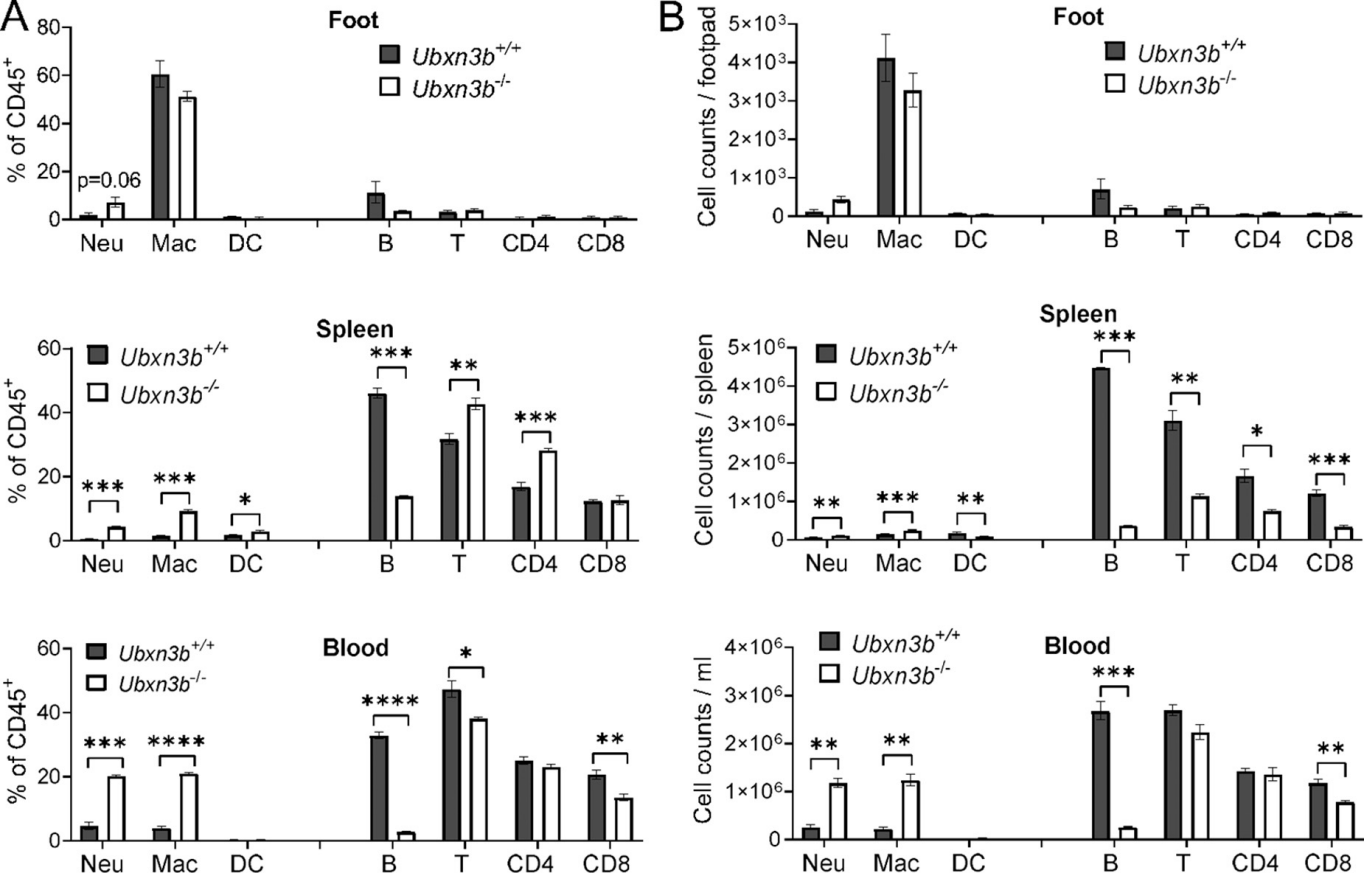
UBXN3B was essential for steady-state immune cell hemostasis. Various immune cell compartments in the foot, spleen, and blood were analyzed by flow cytometry. (A) The percentage relative to CD45^+^ cells, and (B) counts of each immune cell type. Neu, neutrophil; Mac, macrophage; DC, dendritic cell. Each symbol is one mouse. Bar, mean ± SEM; N = 4. *, *P* < 0.05; **, *P* < 0.01; ***, *P* < 0.001; ****, *P* < 0.0001 (two-tailed Student's *t* test). The results are representative of 2 independent experiments.

Because immune infiltration into muscles and joints was a hallmark of CHIKV arthritis, by H&E staining we confirmed that there were much more immune cells in the CHIKV-inoculated *Ubxn3b*^−/−^ than *Ubxn3b*^+/+^ feet (Fig. 1C). We then resolved the different immune compartments in the feet inoculated with CHIKV by flow cytometry. We observed higher ratios and numbers of neutrophils and macrophages, but fewer B and CD4 T cells in the *Ubxn3b*^−/−^ than *Ubxn3b*^+/+^ feet on day 7 pi. Notably, the B cell counts were reduced by ~5-fold. These differences were observed in other CHIKV-target immune organs, e.g., blood and spleens. In the spleen, both CD4 and CD8 T cell populations were also reduced by ~3-fold (Fig. 5A and B). Of note, the neutrophil-to-lymphocyte (N/L) ratios were much higher in all the tissues of the *Ubxn3b*^−/−^ mice (Fig. 5C). These data suggested that UBXN3B signaling was essential for not only viral clearance but also immune cell homeostasis. We then asked if this dysregulated immune system in the *Ubxn3b*^−/−^ mice was induced by CHIKV or constitutive. To this end, we analyzed the steady-state immune compartments. The total immune cell numbers in the feet of both uninfected *Ubxn3b*^−/−^ and *Ubxn3b*^+/+^ mice were low with macrophages (mostly resident macrophages) being the dominant immune population, and there were no significant differences in either the myeloid or lymphoid compartments (Fig. 6). The ratios and counts of neutrophils and macrophages were much higher (4- to 7-fold), while B cells were much lower (3- to 10-fold) in the spleens and blood of *Ubxn3b*^−/−^ than those in *Ubxn3b*^+/+^ animals (Fig. 6). The T cell counts were lower in the spleens of *Ubxn3b*^−/−^ than those in *Ubxn3b*^+/+^ mice. After infection, the numbers of total immune cells and individual compartments in both *Ubxn3b*^+/+^ and *Ubxn3b*^−/−^ feet increased significantly compared to the uninfected *Ubxn3b*^+/+^ and *Ubxn3b*^−/−^ mice, respectively. While the changes (post/prior CHIKV) in neutrophil, B, and T cell counts were comparable between the *Ubxn3b*^+/+^ and *Ubxn3b*^−/−^ mice, the change in macrophage in the *Ubxn3b*^−/−^ mice was 2.5 times as many as in the *Ubxn3b*^+/+^ mice. Nonetheless, the absolute counts of neutrophils and macrophages were significantly greater, while the B cell number was significantly smaller in the infected-*Ubxn3b*^−/−^ than infected-*Ubxn3b*^+/+^ mice (Fig. 5). These data demonstrated that UBXN3B was essential for immune cell homeostasis during steady-state and CHIKV infection.

**FIG 7:**
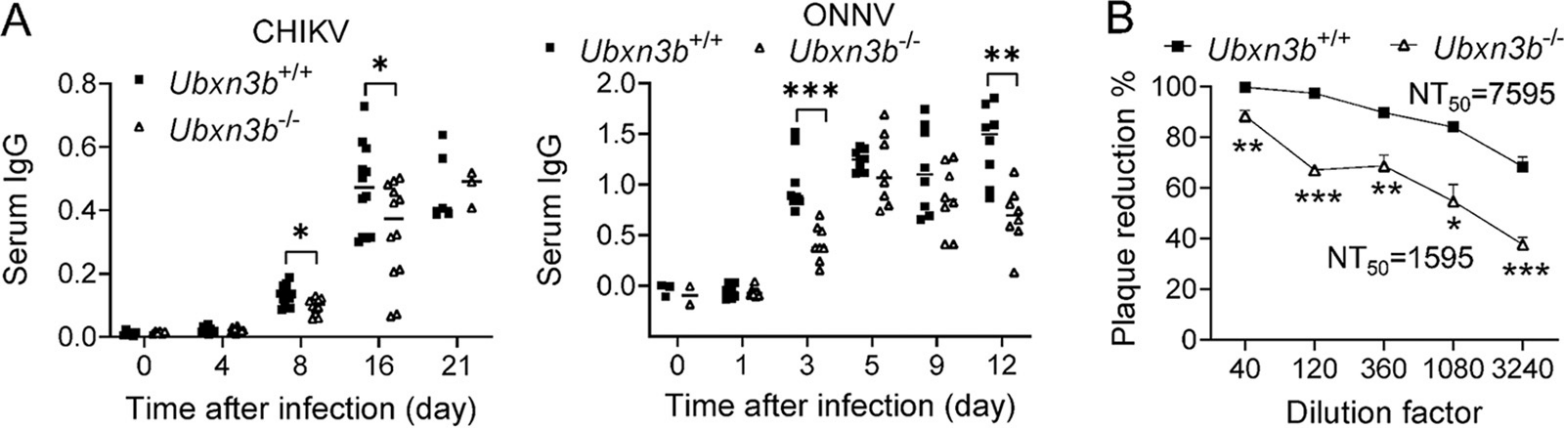
Virus specific-IgG production was impaired in *Ubxn3b*^−/−^ mice. Sex-and-age matched littermates were administered 1 × 10^5^ PFU of CHIKV or 5 × 10^5^ of ONNV subcutaneously in one footpad. (A) The serum IgG levels were quantitated by ELISA and presented as optical density at 450 nm (OD_450_). Each symbol is one animal. The small horizontal line is the median of the result. (B) Percent plaque reduction of day 16 CHIKV sera over a preimmune serum. NT_50_, neutralizing titer of 50%. Preimmune sera assay for day 16 sera. *, *P* < 0.05; ***, *P* < 0.001 (two-tailed Student's *t* test). The results are representative of 2 independent experiments.

Because B lymphocytes were significantly reduced in the *Ubxn3b*^−/−^ mice, we asked if this deficiency influenced the production of virus-specific antibodies. Indeed, the concentrations of serum anti-CHIKV/ONNV immunoglobulin G (IgG) were lower in the *Ubxn3b*^−/−^ than those in *Ubxn3b*^+/+^ mice (Fig. 7A). Moreover, the CHIKV-neutralizing antibody levels in the *Ubxn3b*^−/−^ sera were also reduced. The fold-dilution for neutralizing 50% infectious virions (NT_50_) was 7595 for the *Ubxn3b*^+/+^ sera and 1595 for *Ubxn3b*^−/−^ sera (day 16 pi), suggesting that the titers of CHIKV-neutralizing antibodies in the *Ubxn3b*^−/−^ sera were reduced by ~3.7-fold (Fig. 7B).

### A cell-intrinsic antiviral function of UBXN3B.

**FIG 8:**
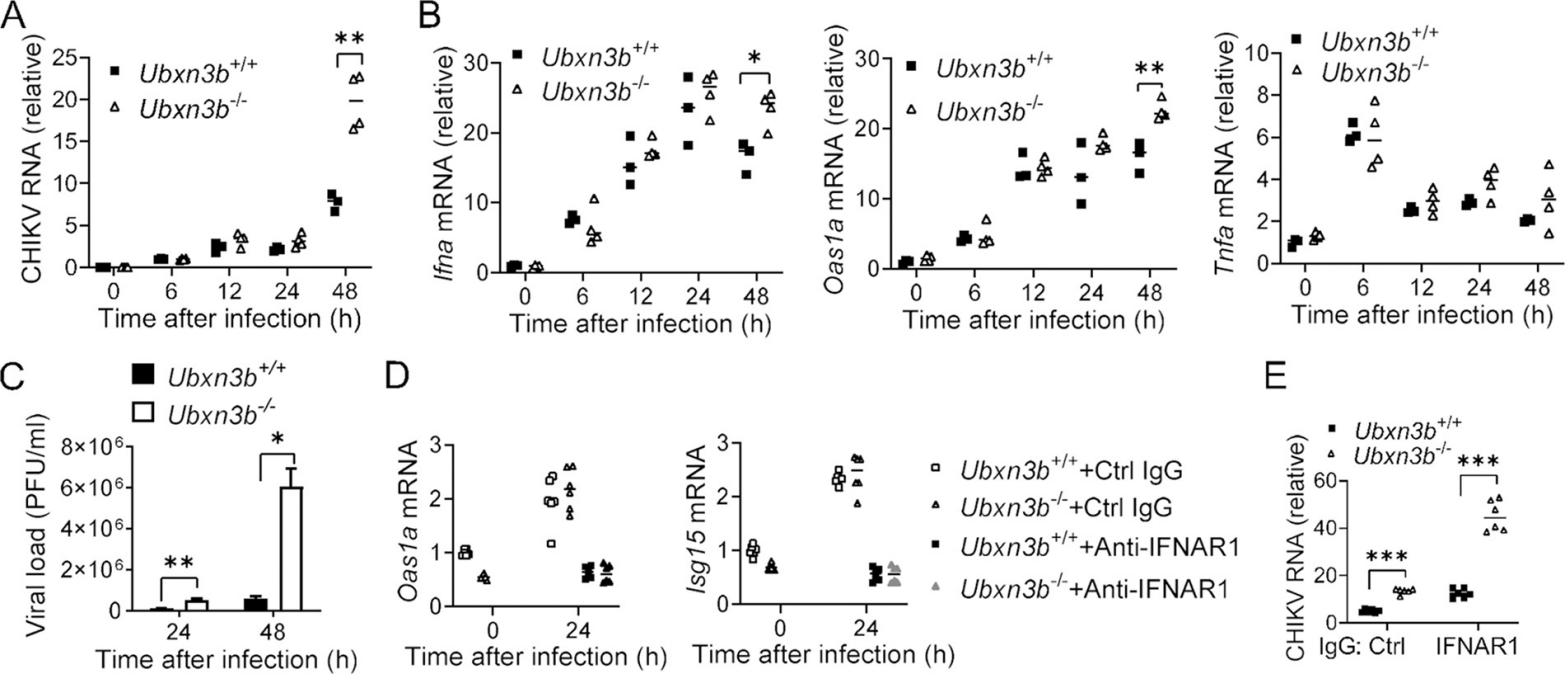
UBXN3B restricted CHIKV infection in an IFN-I-independent manner. (A and B) Bone marrow-derived mouse dendritic cells were infected with CHIKV/ZIKV at a multiplicity of infection (MOI) of 1. Quantitative RT-PCR (qPCR) quantification of (A) viral RNA and (B) immune gene mRNA. Each symbol is one mouse. The small horizontal line is the median of the result. (C) Viral titers (PFU/mL) in the supernatants of mouse embryonic fibroblast (MEFs) infected with CHIKV (MOI = 0.5). Bar, mean ± SEM (N = 3). qPCR quantification of (D) ISG mRNA and (E) CHIKV RNA. MEFs were treated with an anti-IFNAR1 antibody or control IgG (8 μg/mL) for 2 h and then infected with CHIKV (MOI = 0.5) in the presence of antibodies for 24 h (N = 6 biological repeats). *, *P* < 0.05; **, *P* < 0.01 (two-tailed Student's *t* test). The results are representative of 3 independent experiments.

The above-mentioned data demonstrated a deficiency in the B cell compartment and IgG production in the *Ubxn3b*^−/−^ mice, which may underlie delayed viral clearance. However, the *Ubxn3b*^−/−^ mice presented much higher CHIKV viremia as early as at 2 days pi with an intact type, I IFN response (Fig. 2A and B) before the adaptive immune response was activated. We then asked if UBXN3B played a cell-intrinsic antiviral role. To this end, we infected bone marrow-derive dendritic cells (BM-DCs) over 48 h. Skin DCs were one of the early targets of mosquito-transmitted CHIKV in nature and may help CHIKV disseminate systemically. Although CHIKV replication was not productive in DCs, the viral RNA loads in the *Ubxn3b*^−/−^ were ~2-fold higher than those in the *Ubxn3b*^+/+^ cells at 48 h pi (Fig. 8A). The mRNA levels of *Ifna*, *Tnfa*, and *Oas1a* (an interferon-induced gene) in the *Ubxn3b*^−/−^ cells trended slightly higher than those in the *Ubxn3b*^+/+^ cells (Fig. 8B). We confirmed the critical role of UBXN3B in the control of CHIKV replication in a highly permissive cell type, primary mouse embryonic fibroblast (MEF) (Fig. 8C) (37). These results suggested that UBXN3B restricted CHIKV replication independently of type I IFNs. To corroborate this, we examined the role of UBXN3B in CHIKV infection when type I IFN signaling was absent. We used an anti-IFNAR1 antibody to block the type I IFN receptor signaling. We noted that the viral loads in the anti-IFNAR1 antibody-treated cells were higher than those in control IgG-treated cells, and induction of interferon-stimulated genes (*Oas1a*, *Isg15*) was completely abolished suggesting an efficient blockade of IFNAR1 (Fig. 8D). Nonetheless, the viral loads in *Ubxn3b*^−/−^ cells were still higher than those in *Ubxn3b*^+/+^ cells in either situation (Fig. 8E).

## DISCUSSION

Although UBXN3B has been implicated in lipid metabolism (39–46), protein degradation (47, 48), mRNA stability (49), protein translocation (50), and stress granule disassembly (51), its *in vivo* physiological functions remain largely elusive. With a mouse model, we recently demonstrated that UBXN3B regulates STING-mediated innate immunity to a DNA virus (37). In this study, we discovered a critical role of UBXN3B in the control of RNA virus replication and pathogenesis. Similar to *Sting* deficiency (38), *Ubxn3b* deletion led to elevated viremia and exacerbated foot swelling during CHIKV and ONNV infection. However, the cytokine expression remained largely intact or even higher in the *Ubxn3b*^−/−^ mice and DCs. Moreover, viral replication was still enhanced in *Ubxn3b*^−/−^ cells when type I IFN signaling was simultaneously blocked. These results suggested that UBXN3B signaling restricted CHIKV and ONNV replication in a cell-intrinsic manner but independently of type I IFNs. However, both STING and UBXN3B are known to contribute to the induction of type I IFNs by some RNA viruses, including vesicular stomatitis virus (VSV) and Sendai virus (SeV) (37, 52). This discrepancy is likely because VSV and SeV solely depend on RIG-I (53), which can crosstalk with STING to induce type I IFNs (54). CHIKV and ONNV are less reliant on RIG-I and can activate melanoma differentiation-associated protein 5 (MDA5) for a sufficient type I IFN response (55). Mechanistically, STING is known to restrict the translation of RNA viruses (56). Because both UBXN3B and STING localize to the endoplasmic reticulum (ER), it is reasonable to speculate that UBXN3B works together with STING to inhibit RNA virus translation.

Notably, *Ubxn3b* deficiency increased myeloid immune cell (neutrophil, macrophage) numbers, while a reduction in B lymphocytes in the feet, spleens, and blood during CHIKV infection. Neutrophil and B cell ratios were strikingly different between the *Ubxn3b*^−/−^ and *Ubxn3b*^+/+^ mice. Neutrophils infiltrate joints rapidly after CHIKV infection and seem to contribute to acute CHIKV arthritis pathogenesis (57), while B cells are essential for the control of CHIKV replication and arthritis (58). Macrophages constituted the largest immune cell population in feet following CHIKV infection, and its number was moderately higher in the *Ubxn3b*^−/−^ mice. These activated macrophages could be the main cellular reservoir for CHIKV persistence during the late stages of infection and contribute to sustained inflammation (24, 59). On the other hand, heightened immunopathology and tissue damage persisted in the *Ubxn3b*^−/−^ mice even after CHIKV clearance (16 days pi), suggesting that UBXN3B controls CHIKV immunopathogenesis separately from viral replication. Indeed, chronic CHIKV arthritis pathogenesis can progress without active viral replication (60). Thus, more myeloid, and fewer B cells may account for increased viral replication, delayed viral clearance, and exacerbated, and prolonged inflammation in the *Ubxn3b*^−/−^ mice. Strikingly, this aberrant immune system was also observed in the uninfected *Ubxn3b*^−/−^ mice, suggesting an important role of UBXN3B in constitutive hematopoietic homeostasis. This new function is irrelevant to STING as *Sting*-deficient mice have normal immune cell compartments (61). Instead, UBXN3B deficiency might resemble the aging immune state, which is characteristic of low-grade inflammation mainly driven by macrophages and immunosenescence (62).

In humans, severe CHIKV arthritis is associated with elevated serum cytokine/chemokine levels, such as IL-1β, IL-6, TNF-α, CXCL10, etc. (63). Indeed, we noted that the serum CXCL10, IL-6 and TNF-α levels were higher in the *Ubxn3b*^−/−^ than those in *Ubxn3b*^+/+^ mice, consistent with more severe arthritic pathology in the *Ubxn3b*^−/−^ mice. Notably, the CXCL10 levels remained higher even after the viremia clearance at 8 to 16 days after CHIKV or ONNV infection, which is correlated with the prolonged foot inflammation in the *Ubxn3b*^−/−^ mice. CXCL10 is a chemoattractant for monocytes/macrophages/T cells/natural killer cells/DCs and is associated with severe CHIKV arthritis in humans (64). CXCL10 may contribute to alphaviral arthritis pathogenesis by recruiting monocytes/macrophages to the alphavirus-infected mouse feet (65). IL-1β is another established biomarker of CHIKV arthritis severity in humans (66, 67). We also observed an increase in the serum CCL2 and CCL3 levels in the *Ubxn3b*^−/−^ mice. CCL2 and CCL3 are chemoattractants expressed primarily by monocytes and macrophages and essential regulators of neutrophil and macrophage recruitment and function. These data agree with the increase of neutrophil and macrophage populations in the *Ubxn3b*^−/−^ mice. However, we did not detect a significant increase in the serum IL-1β levels throughout CHIKV infection (1 to 16 days) compared with prior infection in all the mice. This might be not surprising because CHIKV infection is much less productive in adult wild-type mice than in humans. However, some studies have demonstrated the induction of IL-1β expression by CHIKV in mice. One study showed that the *Il1b* mRNA expression was upregulated in the ipsilateral feet of adult mice following CHIKV infection (~10^4^ PFU). Another study with 4-week-old mice infected with 10^3^ PFU of CHIKV showed that the IL-1β protein levels in the ipsilateral feet were upregulated too (68). In neonatal mice (8 to 9 day-old), the serum IL-1β levels were upregulated following CHIKV infection (1 × 10^5^ to 2 × 10^5^ PFU) (69, 70). In contrast, no significant serum IL-1β protein was detected during CHIKV infection (1 × 10^4^ PFU) in adult mice (71). These studies demonstrated that, although CHIKV infection in mice upregulated IL-1β expression locally in the ipsilateral feet, whether it can increase the systemic (serum) IL-1β protein level was probably reliant on the animal models and infection conditions of choice.

In summary, our studies suggested that UBXN3B restricted CHIKV/ONNV infection and immunopathogenesis by dual mechanisms, including STING signaling and immune cell homeostasis. The mouse UBXN3B function may be potentially recapitulated in humans because the UBXN3B protein is highly conserved in mammals (98% identity between human and rodent protein). Immediate future work will pinpoint the role of UBXN3B in hematopoietic homeostasis, particularly B cell development.

## MATERIALS AND METHODS

### Mouse models.

The mouse line with the exon 1 of *Ubxn3b* flanked by two LoxP sites (Ubxn3b^flox/flox^) was generated via homologous recombination by Fujimoto at Nagoya University (43). The homozygous *Ubxn3b*^flox/flox^ was then crossed with homozygous tamoxifen-inducible Cre recombinase-estrogen receptor T2 mice (Jackson Laboratories, stock number 008463) to generate Cre^+^ *Ubxn3b*^flox/flox^ littermates. To induce *Ubxn3b* deletion, >6-weeks old mice were injected with 100 μL of tamoxifen (10 mg/ mL in corn oil) (Sigma, number T5648) via intraperitoneal (IP) every 2 days for a total duration of 8 days (4 doses). Successful deletion of *Ubxn3b* was confirmed in our recent study (32). Half of Cre^+^ *Ubxn3b*^flox/flox^ litters were treated with tamoxifen and designated *Ubxn3b*^−/−^. The other half were treated with corn oil only and designated *Ubxn3b*^+/+^. Mice were allowed to purge tamoxifen for at least 4 weeks before any infection or analyses were performed. The animal protocols were approved by the Institutional Animal Care and Use Committee at the University of Connecticut Health Center and Yale University.

### Antibodies, cell lines, and viruses.

The goat anti-mouse IFNAR1 (number AF30369) was purchased from R&D Systems (Minneapolis, MN 55413, USA), rabbit anti-GAPDH (Clone D16H11, number 5174), anti-β-Actin (Clone 13E5, number 4970) and STING (Clone D2P2F, number 13647) were from Cell Signaling Technology (Danvers, MA, USA). Human embryonic kidney 293 cells transformed with T antigen of SV40 (HEK293T, number CRL-3216) and Vero cells (monkey kidney epithelial cells, number CCL-81) were purchased from American Type Culture Collection (ATCC) (Manassas, VA, USA). These cell lines were not listed in the database of commonly misidentified cell lines maintained by ICLAC. Cells were grown in DMEM supplemented with 10% fetal bovine serum (FBS) and antibiotics/antimycotics (Life Technologies, Grand Island, NY, USA). We routinely added MycoZAP (Lonza Group, Basel, Switzerland) to cell cultures to prevent mycoplasma contamination.

The CHIKV French La Reunion strain LR2006-OPY1 was a kind gift of the Connecticut Agricultural Experiment Station in New Haven, CT, USA. The CHIKV vaccine strain 181, clone 25 was recovered from a plasmid and used for *in vitro* infection and enzyme-linked immunosorbent assay (ELISA) (72, 73). The ONNV UgMP30 strain (NR-51661) was provided by BEI Resources. All these viruses were propagated in Vero cells.

### Differentiation of bone marrow DCs, isolation and treatment of embryonic fibroblasts with 4-OH tamoxifen.

Bone marrow-derived DCs were induced from bone marrow cells with 10 ng/mL murine GM-CSF (PeproTech) in RPMI 1640 medium containing 10% (volume/volume [vol/vol]) FBS (Invitrogen, Carlsbad, CA, USA), 100 U/mL penicillin, and 100 μg/mL streptomycin (Invitrogen) for 6 to 8 days at 37°C and 5% CO_2_ (37). Mature BM-DCs were cultured in RPMI 1640 medium overnight and then washed once with the prewarmed fresh medium before use.

Pregnant Cre^+^ *Ubxn3b*^flox/flox^ females were euthanized on days 12 to 14 of gestation. Embryos were decapitated and eviscerated and then digested with trypsin for 10 min at 37°C rotating. Fibroblasts were filtered through a 100 μM filter, cultured in RPMI 1640 medium (Life Technologies, NY, USA), and supplemented with 10% FBS and antibiotics/antimycotics. One-half of the Cre^+^ *Ubxn3b*^flox/flox^ embryonic fibroblasts (MEFs) were treated with 2 μM 4-hydroxyl (OH) tamoxifen for 4 to 5 days to generate the *Ubxn3b*^−/−^ cells. The other half was treated with solvent (dimethyl sulfoxide, DMSO), resulting in *Ubxn3b*^+/+^. After induction, the cells were further passaged three times in RPMI medium without tamoxifen.

### Plaque-forming assay.

Quantification of infectious viral particles in sera, cell culture supernatants, and homogenized tissues were performed on Vero cell monolayers (74). Briefly, viral samples (appropriately diluted) were incubated with confluent Vero cells (6-well plate) at 37°C for 2 h. The inoculum was then removed and replaced with 2 mL of DMEM complete medium with 1% SeaPlaque agarose (catalog number 50100, Lonza). The cells were incubated at 37°C, 5% CO_2_ for 3 days, and on the fourth day, the cells were stained with Neutral Red (Sigma-Aldrich) overnight.

### Mouse infection and monitoring.

About 1× 10^5^ to 5 × 10^5^ plague-forming units (PFU) of CHIKV LR2006-OPY1 or ONNV were inoculated at the ventral side of a hind foot subcutaneously. The thickness and width of the perimetatarsal area of the hind foot (inoculated with the virus) were measured using a precision metric caliper. The foot dimension was calculated as width × thickness, and the results were expressed as the percent increase in the foot dimension after infection (D_n_) over its baseline before infection (D_0_), using the formula: 100% × (D_n_-D_0_)/D_0_.

### Tissue histology.

Tissues were fixed in 4% paraformaldehyde (PFA), embedded in paraffin, cut into 4 μM-thick sections, immobilized to glass slides, decalcified, and processed for hematoxylin and eosin staining. Arbitrary arthritic disease scores (on a 1 to 5 scale with 1 being the slightest and 5 the worst) were assessed using a combination of histological parameters, including exudation of fibrin and inflammatory cells into the joints, alteration in the thickness of tendons or ligament sheaths, and hypertrophy and hyperlexia of the synovium in a double-blinded manner (75).

### Reverse transcription and quantitative PCR.

Up to 1 × 10^6^ cells or 10 mg of tissue was collected in 350 μL of lysis buffer (Qiagen RNeasy Minikit). RNA was extracted following the Qiagen RNeasy manufacturer’s instructions. Reverse transcription of RNA into complementary DNA (cDNA) was performed using the BIO-RAD iScript cDNA Synthesis kit. Quantitative PCR (qPCR) was performed with gene-specific primers and SYBR green PCR master mix. Results were calculated using the –ΔΔCt method and a housekeeping gene, beta-actin, as an internal control. The qPCR primers were reported in our previous studies (38, 65).

### Fluorescence-activated cell sorting.

FACS was performed according to our published study (65). Mouse tissues were minced with a fine scissor and digested in 4 mL of digestion medium (20 mg/mL collagenase IV [Sigma-Aldrich, St. Louis, MO, USA], 5 U/mL dispase [StemCell, Cambridge, MA, USA], and 50 mg/mL DNase I mix [Qiagen, Germantown, MD, USA] in complete RPMI 1640 medium) at 37°C for 4 h. The lysate was filtrated with a 40 μm cell strainer. Cells were then pelleted down by centrifugation at 500 × *g* for 5 min. The red blood cells in the cell pellet were lysed three times with a lysis buffer (catalog number 420301 from Biolegend, San Diego, CA, USA). Cells were suspended in FACS buffer and stained for 30 min at 4°C with the desired antibody cocktails (Biolegend, San Diego, CA, US) of APC-Fire 750-anti CD11b (catalog number 101261, clone M1/70), Alexa Fluor 700-anti Ly-6G (catalog number 127621, clone 1A8), Brilliant Violet 421-anti CD11c (catalog number 117343, clone N418), PerCP-Cy5.5-anti MHC II (catalog number 107625, clone M5/114.15.2), PE-anti Tetherin (PCDA1) (catalog number 12703, clone 10C2), Brilliant Violet 510-anti F4/80 (catalog number 123135, clone BM8), APC-anti CD68 (catalog number 137007, clone FA-11), PE-Dazzle 594-anti CD3 epsilon (catalog number 100347, clone145 to 2C11), Brilliant Violet 711-anti CD4 (catalog number 100557, clone RM4-5), Brilliant Violet 570-anti CD8a (catalog number 100739, clone53 to 6.7), Brilliant Violet 650 anti-CD161 (NK1.1) (catalog number 108735, clone PK136), Zombie UV (catalog number 423107), PE-Cy7-anti CD45 (catalog number 103113, clone 30-F11), TruStain FcX-anti CD16/32 (catalog number 101319, clone 93), Brilliant Violet 421-anti CD45R (B220) (catalog number 103239, clone RA3-6B2), and Alexa Fluor 700-anti CD19 (catalog number 115527, clone 6D5). After staining and washing, the cells were fixed with 4% PFA and analyzed on a Becton, Dickinson FACS ARIA II, CyAn advanced digital processor (ADP). Data were analyzed using the FlowJo software. Among CD45^+^ cells, CD11b^+^ Ly6G^+^ cells were classified as neutrophils, Ly6G^−^ CD11b^+^ F4/80^+^ as macrophages, Ly6G^−^ CD11b^+^ CD115^+^ as monocytes, Ly6G^−^ CD11c^+^ MHC II^+^ as dendritic cells (DC), CD3^+^ as total T cells, CD3^+^ CD4^+^ as CD4 T cells, CD3^+^ CD8^+^ as CD8 T cells, CD19^+^ as B cells.

### Multiplex enzyme-linked immunosorbent assay.

We used a LEGENDPlex (Biolegend, San Diego, CA 92121, USA) bead-based immunoassay to quantify serum cytokine concentrations. The procedures were the same as in the product manual. Briefly, the supernatants or standards were mixed with antibody-coated microbeads in a filter-bottom microplate and incubated at room temperature for 2h with vigorous shaking at 500 rpm. After the removal of unbound analytes and two washes, 25 μL of detection antibodies were added to each well, and the plate was incubated at room temperature for 1 h with vigorous shaking at 500 rpm. Next, 25 μL of SA-PE reagent was then added directly to each well, and the plate was incubated at room temperature for 30 min with vigorous shaking at 500 rpm. The beads were washed twice with wash buffer and then transferred to a microcentrifuge tube. The beads were fixed with 4% PFA for 15 min and resuspended in the assay buffer. The beads were run through a BIORAD ZE5 and the concentrations of analytes were calculated with the standards included in the assay kit using the LEGENDPlex software. The CCL2 (number 88-7391-22) and CCL3 (number 88-56013-22) ELISA kits were purchased from Invitrogen and performed according to the product manual exactly (Waltham, MA, USA).

### Quantification of IgG by ELISA.

Next, ~1 × 10^6^ viral particles in coating buffer (0.05 M carbonate-bicarbonate, pH 9.6) were coated to a 96-well microplate at 4°C overnight. The plate was washed once with wash solution (50 mM Tris, 0.14 M NaCl, 0.05% Tween 20, pH 8.0), and blocked with 4% bovine serum albumin at room temperature for 2 h. Next, 100 μL of each diluted serum (500-fold) was added to a well and incubated at room temperature for 1 h, then washed three times. Then, 100 μL of diluted horseradish peroxidase-conjugated goat anti-mouse IgG was added to each well and incubated at room temperature for 1 h. After stringency wash, 100 μL of substrate 3,3′,5,5′-tetramethylbenzidine (TMB) was added to each well and incubated at room temperature for5 to 30min for color development and terminated by 100 μL of 0.16 M sulfuric acid. The absorption at wavelength 450 nm (A_450_) was read on a Cytation 1 plate reader (BioTek, Winooski, VT, USA).

### Plaque neutralizing assay.

The preimmune and anti-CHIKV mouse sera were heated at 56°C for 20 min and serially diluted in phosphate-buffered saline (PBS). One hundred 20 PFU of CHIKV were mixed with 100 μL of diluted sera, and the mix was incubated at 37°C for 1 h. The mix and fetal bovine serum (FBS)-free DMEM medium was added to Vero cells (see description of the plaque-forming assay for preparation of Vero cells) in a 6-well plate and incubated at 37°C for 4 h. The inoculum was then removed and replaced with 2 mL of DMEM complete medium with 1% SeaPlaque agarose (catalog number 50100, Lonza). The cells were incubated at 37°C, 5% CO_2_ for 3 days, and on the fourth day, the cells were stained with Neutral Red (Sigma-Aldrich) overnight. The dilutions and percent plaque reduction (over control serum) were plotted for polynomial equations, which were used to calculate the neutralizing titer at which viral plaques were reduced by 50% (NT_50_).

### Data availability.

The sample sizes chosen for our animal experiments in this study were estimated according to our prior experience in similar sets of experiments and power analysis calculations (http://isogenic.info/html/power_analysis.html). All animals were included, and no method of randomization was applied. All data were analyzed with GraphPad Prism software by nonparametric or nonparametric two-tailed Student's *t* test depending on the data distribution. *P* ≤ 0.05 was considered statistically significant.
